# Heterogeneous Phase Microwave-Assisted Reactions under CO_2_ or CO Pressure

**DOI:** 10.3390/molecules21030253

**Published:** 2016-02-24

**Authors:** Emanuela Calcio Gaudino, Laura Rinaldi, Laura Rotolo, Diego Carnaroglio, Camillo Pirola, Giancarlo Cravotto

**Affiliations:** 1 Dipartimento di Scienza e Tecnologia del Farmaco and NIS—Centre for Nanostructured Interfaces and Surfaces, University of Turin, Via P. Giuria 9, Turin 10125, Italy; emanuela.calcio@unito.it (E.C.G.); laura.rinaldi@unito.it (L.R.); laura.rotolo@unito.it (L.R.); diego.carnaroglio@unito.it (D.C.); 2 Milestone srl, Via Fatebenefratelli, 1-5, Sorisole 24010, Italy; c.pirola@milestonesrl.com

**Keywords:** microwave-assisted organic reactions, heterogeneous phase, gas pressure, CO carbonylation, CO_2_ insertion

## Abstract

The present review deals with the recent achievements and impressive potential applications of microwave (MW) heating to promote heterogeneous reactions under gas pressure. The high versatility of the latest generation of professional reactors combines extreme reaction conditions with safer and more efficient protocols. The double aims of this survey are to provide a panoramic snapshot of MW-assisted organic reactions with gaseous reagents, in particular CO and CO_2_, and outline future applications. Stubborn and time-consuming carbonylation-like heterogeneous reactions, which have not yet been studied under dielectric heating, may well find an outstanding ally in the present protocol.

## 1. Introduction

The development of environmentally benign and efficient synthetic protocols is still a central goal of current research in chemistry [1,2]. In this regard, heterogeneous multiphase reactions with gaseous reagents in closed microwave (MW) reactors stand out as key alternatives to conventional protocols and provide “greener” chemical conversions. While synthetic chemistry still depends strongly on fossil fuels as an energy source and source of raw materials, the search for renewable raw feedstocks is a prerequisite for sustainable chemical processes [3]. The ambitious aim of decreasing the use of depleting fossil fuel deposits has stimulated an intense research effort in recent years that has been focused on exploiting carbon monoxide (CO) and carbon dioxide (CO_2_) as renewable and inexpensive C_1_ building blocks [4].

Despite their huge potential, CO and CO_2_ are seldom used as reagents in industrial organic synthesis because of the general reluctance to use these gases as reagents and their need for specific pressure-resistant equipment [5]. In particular, CO is an extremely useful carbonyl synthon in organic synthesis. Carbonylation reactions, such as formylation [6], hydroformylation [7], alkoxycarbonylation [8], aminocarbonylation [9], carbonylative Heck [10], Suzuki Miyaura [11], and Sonogashira reactions [12], are industry’s core strategies for converting various bulk chemicals into an extensive set of useful products, such as alcohols, aldehydes and carboxylic acid derivatives. CO works as a high affinity ligand for transition metal catalysts (especially palladium), because of its dual ability to act as a σ-donor and π-acceptor, making “CO chemistry” an easy and practical route for the introduction of a carbonyl group into an organic substrate. Despite the above-mentioned advantages, CO’s reputation as “the silent killer” that leads to asphyxiation (as an oxygen competitor in haemoglobin binding), has generated major concern and warnings concerning its use. A number of protocols that see CO released* in situ* have been developed to circumvent the safety issues surrounding this invisible, odourless, tasteless and highly toxic gas [13,14,15,16,17,18,19]. These methods include the use of CO-equivalents (alkyl formates [20], formic acid, formic anhydride, formamide [21,22,23], *N*-substituted formamide [24], carbamoylstannanes [25], carbamoylsilane [26]), and CO-releasing reagents (metal carbonyls [27]). Unfortunately, these reagents are relatively expensive and sometimes require harsh reaction conditions for CO release (strong base combined with high temperatures and long reaction times).

**Figure 1:**
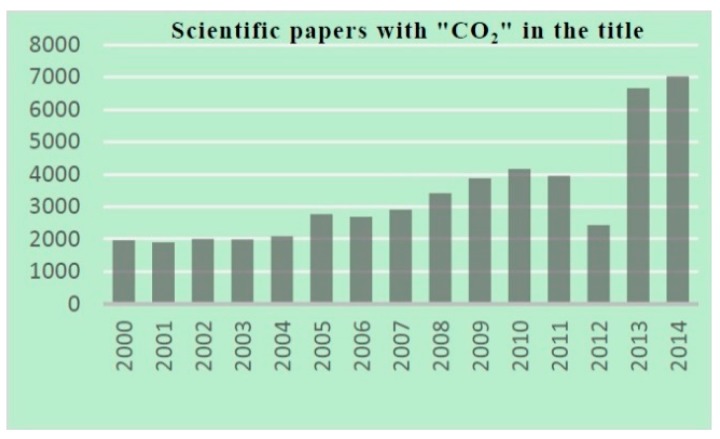
Scopus^®^ (Elsevier) scientific document analysis.

Only 1% of the total amount of CO_2_ on Earth is currently being used by chemical industries, because of its chemical inertness and the high cost of capture and storage [28]. The number of scientific documents that deal with CO_2_ use has grown exponentially over the last decade, with the notable exception of a sudden decrease in 2012, when the Fukushima disaster in Japan also affected the scientific production of that country (Figure 1). The large-scale transformation of CO_2_ into basic bulk chemicals remains a long-standing challenge and fascinating early dream. The insertion of CO_2_ into metal-element bonds is already a common procedure, mainly exploited for the synthesis of fine chemical and a few materials, rather than for a broader use on ton scale [29,30].

In particular, CO_2_ gas plays an important role in the 100% atom economy synthesis of organic carbonates (including cyclic carbonates and polycarbonates) [31,32], urea derivatives [23], oxazolidinones [33], formic acid [34], solar fuels [35] and so forth [36]. However, it should be noted that the carbon atom in CO_2_ is present in its most oxidized state, which results in higher stability and lower reactivity than CO [37]. High energy input is necessary for the sustained use of CO_2_ as a C_1_ environmentally friendly building block and the use of alternative energy source or technologies is thus recommended.

In synthetic organic chemistry, one of the most direct ways to pursue green chemistry is the use of enabling technologies that include heterogeneous phase synthesis as well as relatively new techniques such as MW-assisted synthesis, continuous flow reactors,* etc.* that have been developed to speed up synthetic transformations and achieve process intensification [38,39]. Since the first report in 1986 [40,41], MW-assisted chemistry has fulfilled its promise of being a fast synthetic technique and it has now been recognized as one of the most powerful and sustainable tools in organic chemistry [42]. MW irradiation provides efficient, in-core volumetric reaction mixture heating via the direct coupling of electromagnetic waves with polar entities (solvents, reagents, catalysts), providing rapid energy transfer (less than nanosecond) to the reaction mixture [43]. The efficiency of MW flash heating has resulted in dramatic reductions in reaction times, improvements in product yields and purity, even in heterogeneous gas-phase [44] or in a synergistic combination with other energy sources [45]. Despite MW assisted organic synthesis (MAOS) being a flourishing topic, little attention has been paid to the possibility of combining MW technology with gaseous reagents especially at low pressure (hydrogenation [46], hydroformylation [47], and alkoxy- [48] or aminocarbonylation [49]).

This is feasible because all MW reactors have been designed to resist the pressure generated by solvents used in it and can be considered as small autoclaves that can carry out reactions with pressurized gasses. Only a few reports have dealt with the combined use of CO or CO_2_ in MW at higher gas pressures. The recent introduction onto the market of new pressure resistant (up to 200 bar) high power (up to 1.5 kW) MW reactors equipped with separate multiple gas inlets, which can be considered autoclaves, could pave the way for heterogeneous gas-phase MW reactions [50]. This review aims to summarize recent work in this area and evoke some potential applications, including in the field of flow techniques thanks to the recent development of commercial MW flow reactors for organic synthesis under pressure [51,52,53,54].

## 2. MW-Assisted Reactions under CO_2_ Pressure

As stated above, only a limited number of studies into the MW-assisted synthesis of fine chemicals have been reported in the literature. This is normally due to the instrumental difficulty of performing MW reactions under gas pressure. The synthesis of cyclic and linear carbonates is the most common application of MW. The synthesis of five-membered ring carbonates from epoxides and CO_2_ is a promising methodology from a resource utilization standpoint because of the 100% atom economy of the reaction and the wide variety of applications of cyclic carbonates, which include use as polar aprotic solvents, precursors of polymeric materials and intermediates in the syntheses of pharmaceuticals [55].

In 2004, Nüchter* et al.* [56] described the first examples of the MW-assisted reactions of different oxiranes with CO_2_ in ionic liquids (ILs). Being highly polar, ILs are rapidly heated by MW irradiation [57] affording in shorter reaction times comparable yields (Table 1). Work-up procedures have been explained in detail and help to envisage the scale-up of the process. Compared to conventional conditions, these reactions are much faster, with higher energy efficiency and precise parameter monitoring and control. MW heating has been used in one of the most active catalytic systems for CO_2_ coupling with epoxides; namely zinc salts in ILs [58]. Zinc phenosulfonate octahydrate and C_4_NBr efficiently catalysed the solvent-free synthesis of cyclic carbonates with remarkable turnover frequency (TOF). Park and co-workers [59,60] have proposed novel silica-supported ILs for the cycloaddition of epoxides and CO_2_ under MW irradiation as a means to overcome the major drawback of IL catalysis, their recovery from the reaction mixtures and recycling.

Heterogeneous catalysis is generally preferred by the chemical industry as it leads to easier separation and the possible use of a fixed-bed reactor. However, supported catalysts suffer from the limited diffusion of reagents and products between the solution and the catalyst surface. MW irradiation can nevertheless strongly enhance activity via the selective overheating of catalyst particles [61]. The rapid and selective MAOS of cyclic carbonates has been established using homogeneous (Entry 3, Table 1) and heterogeneous IL (Entry 4, Table 1) catalysts. Both catalysts showed excellent activity and short reaction times (15–30 min) under relatively low CO_2_ pressures (9.7 bar). Unlike conventional reactions, MW technology was able to selectively provide cyclic carbonates rather than mixtures of cyclic and linear products. Even higher selectivity towards the cyclic carbonates was achieved by supporting ILs on silica. This solid catalyst was recycled three times without any significant loss in activity.

An environmentally-friendly synthesis of cyclic carbonates from CO_2_ and epoxides with a HCOOH/KI catalytic system that exploits the synergistic effect between KI and hydrogen bonding donors has been studied under MW irradiation (Table 1, Entry 5) [62]. The search for more efficient processes has prompted Park [63] to propose the use of pyridinium-based ILs as an efficient catalyst for the rapid, solvent-free MW-assisted cycloaddition to yield cyclic carbonates under moderate reaction conditions (Entry 6, Table 1). The cycloaddition reaction occurred over a short reaction time (30 s), resulting in a high TOF which ranged from 200 to 7000 h^−1^. Moreover, the catalyst was recovered and reused three times without losing its catalytic properties.

**Scheme 1:**
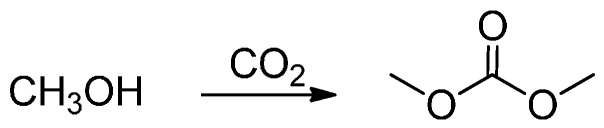
Dimethyl carbonate synthesis.

The second interesting field of use for CO_2_ is the synthesis of dimethyl carbonate (DMC, Scheme 1).

Direct synthesis of DMC from CO_2_ and methanol is a very attractive reaction, not only because the product is a well-known low polluting substitutive intermediate of toxic phosgene or dimethyl sulfate, but also because CO_2_ can be consumed as a greenhouse compound in this process. In 2001, the first example of this synthesis was reported as a facile method for the quick synthesis of DMC from CO_2_ and methanol under MW irradiation [64] and it was compared with conventional heating. In that work, rapid MW heating resulted in higher selectivity to DMC, while conductive heating (slow heating rate) may favour the formation of DMC. Another MW-promoted DMC synthesis reaction with methanol and CO_2_ was carried out over a K_2_CO_3_/CH_3_I catalyst in the presence of IL by Du* et al.* (2012) [65]. IL may affect the polarity of the intermediates and the dipole moment at the transition state, by which selective MW energy absorption and CO_2_ solubility in the reaction medium were increased. IL may also enhance mass transfer by increasing molecular collisions. Computational studies confirmed the hypothesis that the kinetic and thermodynamic factors driving DMC synthesis may be affected by IL that effectively activate CO_2_ molecules by altering the angle and length of the C=O bond. Such activation facilitates the base-catalyzed insertion step by means of K_2_CO_3_ without affecting the total reaction mechanism.

The last example of MAOS under CO_2_ pressure is a new, green and efficient procedure to obtain isocyanates and urea libraries [66]. A versatile pressure-resistant reactor gave a fast reaction with poor reagent excess. An optimized protocol for a Staudinger-aza-Wittig reaction with polymer bound diphenylphosphine (PS-PPh_2_) under CO_2_ pressure was carried out. This study also synthesized a series of symmetric and asymmetric alkyl/aryl urea derivatives from alkyl bromides in a one-pot procedure.

## 3. MW-Assisted Reactions under CO Pressure

**Scheme 2:**
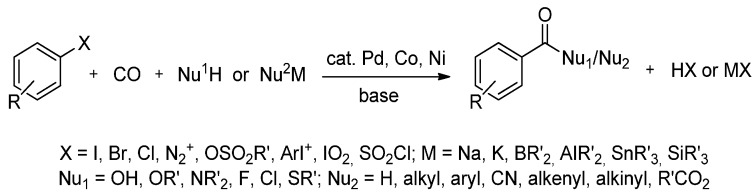
Carbonylation of aryl-X compounds.

Palladium-catalyzed C-C bond formation with aryl halides is currently one of the most important organometallic reactions in synthetic organic chemistry [67]. It offers the practical preparation of a broad spectrum of organic derivatives from simple, commercially available building blocks (Scheme 2). In particular, the carbonylation of aryl halides offers a one-step route to a number of important products, including carboxylic acids, esters and amides [68].

When transferring this chemistry from conventional to dielectric heating a number of critical issues had to be considered to circumvent the difficulties of working in a multiphase gas/liquid (solid) system. Up to fifteen years ago, the lack of technological progress in the design of high pressure resistant MW reactors, resulted in a scarcity of synthetic reports on this topic. Most of the commercially available MW reactors had a pressure limit of 20–30 bar, with no possibility of a prior gas pressurization. The latest generation of pressure-resistant MW reactors may meet all these requirements. Thus, Leadbeater and Kormos have published the first report on the MW-promoted hydroxy- and alkoxycarboxylations of aryl iodides in the presence of Pd(OAc)_2_ as a catalyst using heavy-walled quartz reaction vessels that are pre-pressurized with CO. The authors described a MW-promoted hydroxycarbonylation of aryl iodides in water [69]. Reactions were performed at 165 °C (CO 14 bar) using Pd(Ac)_2_ as a catalyst (0.01 mol % or 1 mol % loadings) in the absence of a phase transfer agent. This is a valid alternative to the use of solid CO sources, giving the corresponding benzoic acids in good yields (59%–80%) inside 20 min, including the ortho-substituted analogues.

Furthermore, Iannelli* et al.* [70], focused their attention on the use of simple ligandless palladium complexes as catalysts in carbonylation chemistry as they described the ethoxycarbonylation of iodobenzene on a 1 mol scale. Six different fast ethoxycarbonylation reactions of several aryl iodides were performed simultaneously using a near-stoichiometric loading of CO gas (1.08 stoichiometric equiv.) in a sealed multimode MW reactor. Excellent conversions (91%–99%) were achieved under MW irradiation in only 30 min at 125 °C on both 50 mmol and 1 mol reaction scales using Pd(Ac)_2_ as a catalyst.

Besides the obvious safety concerns, a large excess of highly toxic CO gas may affect the efficiency of palladium catalysts because of the strong π-acceptor character of CO as a ligand. Furthermore, the rate of oxidative addition is greatly reduced and the clustering of palladium atoms is facile in the presence of CO, leading to non-active palladium species. To overcome these drawbacks, a palladium mediated alkoxycarbonylation procedure using near-stoichiometric quantities of CO gas was also presented by Kormos and Leadbeater [71]. They performed the alkoxycarbonylation reactions using CO in a close to stoichiometric ratio, under MW heating which, as well as the inherent advantages of rate acceleration, offers a convenient method for loading vessels with gas in a safe manner. Precise CO pressure could be monitored in real time by means of a pressure sensor directly placed on the top of the reaction vessel in the monomode reactor. They successfully performed a series of carbonylations with Pd(OAc)_2_ in alcohols (MeOH, *i*-PrOH and *t*-BuOH) at 125 °C under CO (0.97 bar)/N_2_ (9.38 bar) pressure in 20 min. The same authors described the alkoxycarbonylation of several aryl iodides to the corresponding esters using primary, secondary and, the less reactive, tertiary alcohols [72]. These reactions were still performed at 125 °C using 0.1 mol % of Pd(OAc)_2_ as the catalyst and DBU as the base with good to excellent yields in 20–30 min. It is worth noting that the conditions for alkoxycarbonylation are milder than those previously reported by the same authors [62], due to the greater solubility of CO in ethanol than in water.

**Scheme 3:**
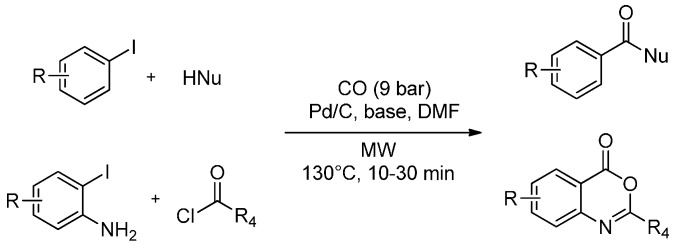
MW-assisted carbonylation and cyclocarbonylation of aryl iodides under ligand-free heterogeneous catalysis.

Heterogeneous catalysts such as Pd/C strongly absorb MW energy, and the local temperature on the catalyst surface is much higher than the bulk temperature measured in the solvent (using optical-fiber thermometers or thermocouples) particularly in the case of low tanδ solvents [73]. The hot catalyst surface may dramatically accelerate chemical reactions when an efficient mass transfer is provided. At this regards, a MW-assisted carbonylation under heterogeneous catalysis with Pd/C has been reported by Salvadori* et al.* [74]. Alkoxycarbonylations were performed using stoichiometric amounts of different primary and secondary alcohols in DMF in the presence of CO gas (9 bar), Pd/C as catalyst and DBU as the base. In 10–20 min at 130 °C, they achieved 75%–99% product yields. Analogously, iodobenzene, CO and amines were transformed into the corresponding amides in good yields (75%–95%) after simple filtration to remove the heterogeneous catalyst. Pd/C was recycled twice without relevant differences being noted. The versatility of Pd/C as a heterogeneous catalyst for the carbonylation reaction is also confirmed by the possibility of continuing the MW-assisted cyclohydrocarbonylation of *o*-iodoaniline with acyl chlorides, producing benzoxazinones in satisfactory yields (70%–82%) with CO gas (9 bar) (Scheme 3).

**Scheme 4:**
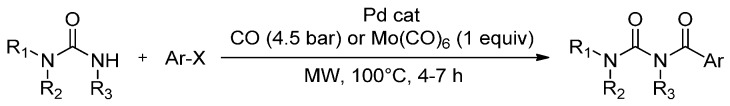
Synthesis of aryl and heteroaryl *N*-acylureas via MW-assisted palladium catalysed carbonylation.

Liptrot* et al.* [75] has reported the first practical synthesis of aryl and heteroaryl *N*-acylureas via the MW-assisted palladium-catalyzed carbonylation of aryl or heteroaryl halides in the presence of urea nucleophiles (Scheme 4). The reactions tolerate a wide range of substituted ureas and substituted aryl or heteroaryl halides while proceeding in good to excellent yields (55%–90% at 100 °C in 4 h), when using either CO (4.5 bar) or molybdenum hexacarbonyl (Mo(CO)_6_) as the CO source in pre-pressurised or standard MW vials. To illustrate the usefulness of this method, the authors also reported the one-step synthesis of diflubenzuron (an important insecticide) in acceptable yield (45%) using PdCl_2_ (dppf)CH_2_Cl_2_ as catalyst.

**Scheme 5:**
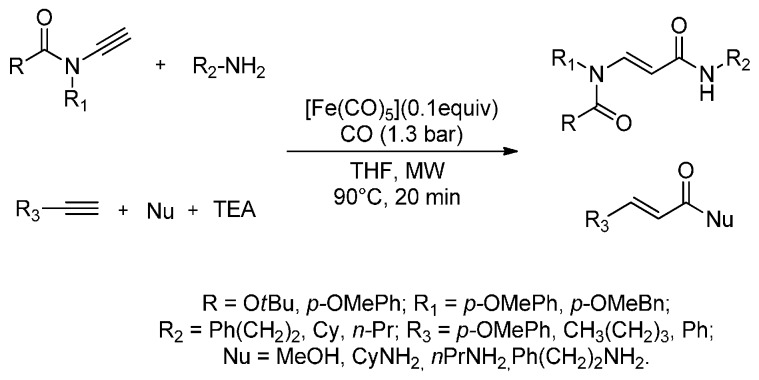
MW-assisted iron-catalysed monocarbonylation of ynamides and terminal alkynes with different nucleophiles.

There has been a recent increase in interest in MW carbonylation reactions that combine the use of CO, as a benign carbon atom source, and new eco-friendly catalysts. In fact, Pizzetti* et al.* (2011) [76] have developed the first catalytic MW-assisted aminocarbonylation of ynamides using [Fe_3_(CO)_12_] as the catalyst precursor and TEA as the only ligand. The aminocarbonylation products were obtained in only 20 min of irradiation (90 °C and 250 W) in good to excellent yields (67%–90%). The authors described an atom-efficient aminocarbonylation procedure in which all reactants were used in a stoichiometric amount under low CO pressure (1.3 bar). The same MW procedure was easily applied to the alkoxycarbonylation of terminal alkynes to give a new class of (*E*)-acrylamides that was regioselectively obtained in only 20 min (44%–78% yields). Furthermore, this procedure was easily exploited for the synthesis of acryl esters from ynamides and alkynes when using MeOH as nucleophile (Scheme 5). MW irradiation allowed milder reaction conditions to be used; lower temperatures and gas pressures and shorter reaction times.

**Scheme 6:**
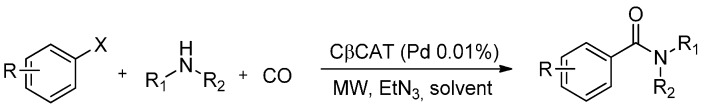
MW assisted aminocarbonylation using CβCAT.

The safe and synergistic use of CO in a closed MW reactor has led to a technological breakthrough in aminocarbonylation reactions. To the best of our knowledge, very few reports have dealt with the use of CO gas as an aminocarbonylation reagent inside a MW oven. In this regard, Calcio Gaudino* et al.* [77] have described a fast MW-assisted Pd(II)-catalysed protocol for the synthesis of amides using CO gas (Scheme 6).

Their investigation dealt with the optimization of a synthetic heterogeneous aminocarbonylation protocol which made use of CO gas and a supported palladium catalyst (CβCAT) which was based on Pd(II)-triphenylphosphine embedded in cross-linked β-cyclodextrins (β-CD). Several amide derivatives were synthesized in good yields (59%–95%) in acetonitrile using an aryl iodide/nucleophilic amine ratio of 1:2 and a CO/N_2_ mixture in a MW reactor equipped with a multiposition rack for simultaneous reactions and fine pressure control. The green β-CD based catalyst showed excellent performance, including easy recyclability and no significant loss in activity aside from negligible entrapped metal catalyst leaching.

## 4. A Promising Future for the Use of MW-Assisted Protocols under CO_2_ or CO Pressure

In this section, we report a few relevant examples of reactions that could potentially gain significant advantages from MW-assisted heterogeneous phase protocols under CO_2_ or CO pressure. Available published data leads to expectations of striking increases in reaction rate in all cases.

As mentioned above, CO_2_ plays a pivotal role as a reagent in many fields of modern chemistry. The use of CO_2_ and waste biomass as a chemical feedstock would provide us with a sustainable basis for the future of the chemical industry, if we could balance the high energy requirements of dielectric heating.

**Scheme 7:**
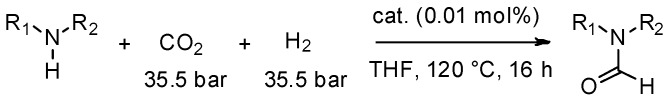
Catalyzed *N*-formylation of amines with H_2_ and CO_2_.

The production of formamides using a MW-autoclave is an eloquent example of potential applications. An alternative green route to the *N*-formylation of amines is the use of cheap, abundant and safe CO_2_ along with H_2_ as a formylating reagent [78]. Zhang* et al.* [79], have recently proposed a highly efficient catalyst for the *N*-formylation of various amines with CO_2_ and H_2_. This catalyst is based on ruthenium-pincer-type complexes and gives the corresponding formamides with excellent yield and selectivity (Scheme 7).

**Scheme 8:**
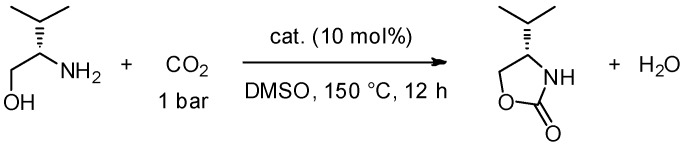
Synthesis of oxazolidinone from β-amino alcohols.

Another hot topic at the moment is the development of new materials that are able to capture CO_2_. With this aim in mind, Takada* et al.* [80], have set up a new procedure for the synthesis of optically pure oxazolidinones from CO_2_ and β-amino alcohols that are derived from natural amino acids (Scheme 8).

The synthetic protocol reported here is simple, straightforward and can be easily replicated. The only limits of this reaction are the harsh conditions and long reaction time. Irradiation with MW under CO_2_ pressure may dramatically simplify the reaction outcome.

**Scheme 9:**
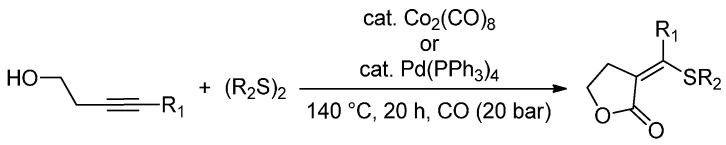
Thiolative lactonization of internal alkynes bearing a hydroxyl group with CO and organic disulfides catalyzed by transition-metal complexes.

The transition-metal-catalysed simultaneous insertion of heteroatom functional groups and CO into organic molecules is one of the most important tools for the direct synthesis of heteroatom-functionalized carbonyl compounds. Dicobalt octacarbonyl and palladium complexes [Pd(PPh_3_)_4_ and Pd(OAc)_2_] were found to exhibit excellent catalytic activity for the thiolative lactonization of internal alkynes, that bear a hydroxyl group, in the presence of CO and organic disulfides [81]. The authors successfully prepared γ-lactone derivatives (71%–98% yields) that bear *exo*-methylene and thio groups with excellent regio- and stereoselectivities (140 °C for 20 h) (Scheme 9). The procedure can also be applied to δ-lactone synthesis. The obtained thiolated lactone derivatives are promising synthetic intermediates, as the thio group can be displaced by a variety of nucleophiles, while exo-methylene groups make them a possible substrate for Michael additions.

Porous organic polymers (POPs) have recently emerged as metal catalyst supports. In particular, Pd supported on triphenylphosphine-functionalized microporous knitting aryl network polymers (Pd@KAPs(Ph-PPh_3_) have attracted much attention. In this context, Lei* et al*. [82] have reported the efficient alkoxycarbonylation of aryl iodides with alcohols and phenols using this supported palladium catalyst in the presence of low pressure CO (1 bar). The corresponding alkoxycarbonylation products were obtained in moderate to excellent yields (74%–96%) at 80 °C in 6–10 h (Scheme 1). The Pd@KAPs(Ph-PPh_3_) catalyst was easily separated by simple filtration and recycled up to ten times without a significant decrease in activity being observed. The salient features of this protocol are the simplicity in catalyst handling, low CO pressure, negligible palladium leaching and good catalyst recyclability.

**Scheme 10:**
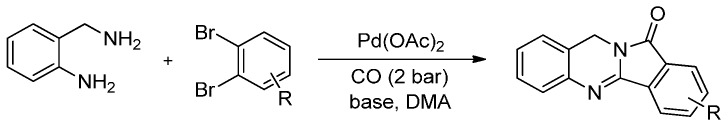
Pd-catalysed carbonylative synthesis of isoindoloquinazolinones.

Pd-catalyzed carbonylation reactions have already become practical tools for the modern organic chemist and offer promising options for the preparation of carbonyl containing compounds. By incorporating one or even more molecules of CO into the parent structure, a carbon chain can be easily increased and the resulting products ready for further modification, which is a factor that holds its own importance. The advantages of carbonylation reactions mean that it is even more attractive to use carbonylations in the synthesis of biologically active heterocyclic compounds. In fact, an interesting and convenient procedure for the carbonylative synthesis of isoindoloquinazolinones was first described by Chen* et al.* [83]. They performed the carbonylative reactions in the presence of Pd(AcO)_2_ at 120 °C from 1,2-dibromobenzenes and 2-aminobenzyl amine, which are readily available substrates. They isolated the isoindoloquinazolinone products in good yields (48%–84% in 16 h), while two CO molecules were installed (Scheme 10).

**Scheme 11:**
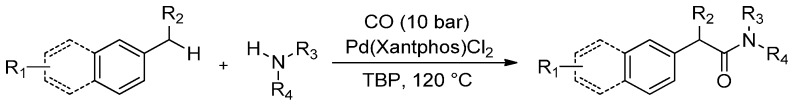
Palladium-catalysed oxidative aminocarbonylation via C-H activation.

The development of new synthetic strategies that work via direct C(sp^3^)-H bond functionalization is a hot topic in organic chemistry. It has recently been established that Pd(Xantphos)Cl_2_ is able to catalyse alkyl aromatic carbonylation to afford the arylacetic acid esters via C-H activation. In this context, Xie* et al.* [84] have reported a new synthetic route for arylacetamides based on C-H activation via a Pd-catalysed oxidative aminocarbonylation of alkyl aromatics with different amines in the presence of CO gas via C(sp^3^)-H activation (Scheme 11).

To further explore the synthetic potential of this process, a series of alkyl aromatics were subjected to the reaction under optimized reaction conditions (CO 10 bar, 120 °C for 16 h). While substrates that bear electron-rich substituents on the aryl ring furnished the corresponding products in high yields (82%–85%), those containing an electron-withdrawing group were less reactive and afforded the desired amides in lower yields (52%–74%). It is worth noting that the tolerance of the halogens on the aromatic ring in this transformation offers an opportunity for subsequent cross-coupling, which facilitates the expedient synthesis of complex arylacetamides.

**Scheme 12:**
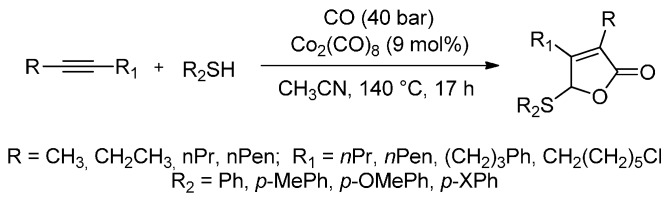
A highly selective cobalt-catalysed carbonylative cyclization of internal alkynes with CO and organic thiols.

Transition-metal-catalysed carbonylations with the simultaneous introduction of a heteroatom are one of the most important tools for the synthesis of carbonyl compounds that bear heteroatom functional groups. Higuchi* et al.* [85] have proved that cobalt carbonyl (Co_2_(CO)_8_) is an excellent catalyst for the carbonylative cyclization of internal alkynes with CO. When Co_2_(CO)_8_-catalyzed reactions of internal alkynes with organic thiols are conducted in acetonitrile under CO pressure (4 MPa), the thiolative lactonization of internal alkynes successfully takes place in 17 h and two molecules of CO are incorporated at 140 °C (Scheme 12). Moreover, this carbonylation provides a useful tool with which to prepare the corresponding α,β-unsaturated γ-thio-γ-lactones (butenolide derivatives) in good yields (50%–70%). In the case of unsymmetrical alkynes, such as 2-octyne and 6-methyl-2-heptyne, thiolative lactonization proceeds with moderate regioselectivity to give the butenolide derivatives whose carbonyl group preferentially bonds to the less hindered acetylenic carbon. The present carbonylation of internal alkynes will open up new routes to the cobalt-catalysed reactions of heteroatom compounds with internal alkynes.

**Scheme 13:**
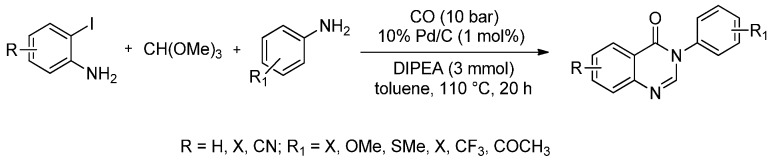
Palladium-catalysed carbonylative coupling of 2-iodoanilines with amines and trimethyl orthoformate.

Most synthetic procedures for quinazolinone derivatives rely on the use of anthranilic acid or its derivatives as the starting materials and generally suffer from low yields, multistep reactions or relatively harsh reaction conditions. The use of Pd/C as a heterogeneous catalyst for the carbonylation of 2-iodoanilines in the presence of trimethyl orthoformate and different amines was first proposed by Natte* et al.* [86]. Excellent yields of 4(3*H*)-quinazolinones were reported (84%–96%) using this four-component reaction approach (110 °C, 20 h) in presence of CO (10 bar) (Scheme 13).

The novelty of this procedure is that it avoids the use of expensive phosphine ligands as well as having the additional advantage of catalyst recovery. Furthermore, five new quinazolinone scaffolds that contain the trifluoroethyl group were introduced using this procedure and gram scale experiments were successfully reported as well.

This short overview highlights the broad scope of the potential applications of MW-assisted protocols under CO pressure in batch mode. Even though several CO and CO_2_ gas-liquid transformations have been optimized in flow-through reactors under conventional heating [87], so far MW flow chemistry remain a rather unexplored field of investigation [53]. This challenging technique should provide valuable guidelines for the scale-up of greener gas-liquid synthetic reactions procedures.

## 5. Conclusions

The conversion of CO_2_, an abundant renewable carbon reagent, and CO into chemicals of academic and industrial interest is of crucial relevance to creating a higher degree of sustainability in chemical processing and production. Moreover, the intensification of chemical processes, which means improving their efficiency and cutting down on energy consumption, requires increased automation and the use of non-conventional energy sources, as well as new, efficient and scalable protocols that can be implemented in continuous-flow reactors. This review aims to indicate some solutions, such as the combined use of gas and MW activation that will have potential applications in the near future of newly-born MW flow reactors.

## Figures and Tables

**Table 1:** MW-assisted synthesis of cyclic carbonates.

Entry	Catalytic System	MW (Watt)	Temp. (°C)	CO_2_ (bar)	Time (min)	Yield (%)	Conv. (%)	Ref.
1	[C_6_MIm]Br (0.05 mol %)	/	140	10	60	86	97	[56]
2	ZnPO (0.043 mol %), C_4_NBr (2.174 mol %)	300	120	30	60	97	/	[58]
3	TABC (0.027 mol %)	200	120–160	9.7	30	91.4	/	[59]
4	SiO_2_/C_4_ImBr (4 wt %)	100	120–160	9.7	15	87.9	/	[59]
5	HCOOH (0.145 mol %), KI (0.016 mol %)	200	/	9.6	15	59	60	[62]
6	C_4_PyBr (0.054 mol %)	200	0-180	9.6	0.5	67.2	70	[63]
